# Proton Pencil Beam Scanning Facilitates the Safe Treatment of Extended Radiation Targets for Hodgkin Lymphoma: A Report from the Proton Collaborative Group Registry

**DOI:** 10.3390/cancers16152736

**Published:** 2024-08-01

**Authors:** Maryam Ebadi, Mark Pankuch, Sean Boyer, John Chang, Craig Stevens, Matthew D. Hall, Shaakir Hasan, James E. Bates, Stella Flampouri, Adam J. Kole, Pranshu Mohindra, Carl Rossi, Parag Sanghvi, Lisa McGee, Zaker Rana, Yolanda D. Tseng

**Affiliations:** 1 Department of Radiation Oncology, University of Washington and Radiation Oncology Division, Fred Hutch Cancer Center, Seattle, WA 98195, USA; mebadi@uw.edu; 2 Northwestern Medicine Proton Center, Warrenville, IL 60555, USA; mark.pankuch@nm.org (M.P.); sean.boyer@nm.org (S.B.); 3 The Oklahoma Proton Center, Oklahoma City, OK 73142, USA; john.chang@okcproton.com; 4 Department of Radiation Oncology, Corewell Health William Beaumont University Hospital, Royal Oak, MI 48073, USA; craig.stevens@beaumont.edu; 5 Miami Cancer Institute, Baptist Health South Florida, Miami, FL 33176, USA; matthewha@baptisthealth.net; 6 New York Proton Center, New York, NY 10035, USA; shasan@nyproton.com; 7 Emory University Hospital Midtown, Atlanta, GA 30308, USA; james.edward.bates@emory.edu; 8 Emory School of Medicine, Atlanta, GA 30322, USA; stella.flampouri@emory.edu; 9 Department of Radiation Oncology, University of Alabama at Birmingham, Birmingham, AL 35294, USA; akole@uabmc.edu; 10 University Hospitals Seidman Cancer Center, Cleveland, OH 44106, USA; 11 California Protons Cancer Therapy Center, San Diego, CA 92121, USA; carl.rossi@californiaprotons.com; 12 Department of Radiation Medicine and Applied Sciences, UC San Diego School of Medicine, La Jolla, CA 92093, USA; psanghvi@health.ucsd.edu; 13 Department of Radiation Oncology, Mayo Clinic, Phoenix, AZ 85054, USA; mcgee.lisa@mayo.edu; 14 Maryland Proton Treatment Center, Baltimore, MD 21201, USA; zaker.rana@umm.edu

**Keywords:** extended target radiation therapy, pencil beam scanning, proton beam therapy

## Abstract

**Simple Summary:**

Using the Proton Therapy Collaborative group registry, we describe the impact of pencil beam scanning (PBS) proton beam therapy (PBT) for Hodgkin lymphoma patients who require radiation to both mediastinal and axillary targets. We performed dosimetric comparisons between delivered PBS plans and photons with butterfly volumetric arc therapy (BVMAT). Compared to BVMAT, PBS had greater target coverage, better conformity, and lower dose heterogeneity while achieving a lower dose to the lungs and heart. Nearly half of the BVMAT plans exceeded the lung V5 constraint (>55%). PBS was well tolerated without any pneumonitis or grade 3+ acute toxicity. We show here that patients requiring both mediastinal and axillary treatment may be another subgroup of lymphoma patients who may benefit from PBT.

**Abstract:**

Because proton beam therapy (PBT) can lower the dose of radiation to the heart, lungs, and breast, it is an established radiation modality for patients with Hodgkin lymphoma (HL). Pencil beam scanning (PBS) PBT facilitates the treatment of more extensive targets. This may be especially of value for lymphoma patients who require RT to both mediastinal and axillary targets, defined here as extended target RT (ETRT), given the target distribution and need to minimize the lung, heart, and breast dose. Using the Proton Collaborative Group registry, we identified patients with HL treated with PBT to both their mediastinum and axilla, for which DICOM-RT was available. All patients were treated with PBS. To evaluate the dosimetric impact of PBS, we compared delivered PBS plans with VMAT butterfly photon plans optimized to have the same target volume coverage, when feasible. Between 2016 and 2021, twelve patients (median 26 years) received PBS ETRT (median 30.6 Gy (RBE)). Despite the large superior/inferior (SI, median 22.2 cm) and left/right (LR, median 22.8 cm) extent of the ETRT targets, all patients were treated with one isocenter except for two patients (both with SI and LR > 30 cm). Most commonly, anterior beams, with or without posterior beams, were used. Compared to photons, PBS had greater target coverage, better conformity, and lower dose heterogeneity while achieving lower doses to the lungs and heart, but not to the breast. No acute grade 3+ toxicities were reported, including pneumonitis. Proton ETRT in this small cohort was safely delivered with PBS and was associated with an improved sparing of the heart and lungs compared to VMAT.

## 1. Introduction

Proton beam therapy (PBT) is an established radiation modality for patients with Hodgkin lymphoma (HL), given prior studies demonstrating its lower doses to the heart, lungs, and breast [1,2] compared to advanced photon techniques, especially among patients with lower mediastinal involvement [3]. In 2018, the International Lymphoma Radiation Oncology Group (ILROG) guidelines highlighted three patient populations that may benefit most from PBT [4]: (1) those with lower mediastinal disease, where the disease spans below the origin of the left main stem coronary artery; (2) young females who are at an increased risk of secondary breast cancer; and (3) those who have been heavily pretreated and are more prone to radiation-related toxicity.

Notably, contemporary guidelines do not comment on whether PBT may also be of benefit for mediastinal lymphoma patients with concurrent axillary disease, defined here as extended target RT (ETRT). These extended targets present additional dosimetric challenges, often limiting the ability to safely deliver consolidation RT by standard photon-based techniques. In a retrospective dosimetric comparative study of protons and photons with butterfly volumetric arc therapy (BVMAT), PBS was associated with a lower mean lung dose, which was associated with a lower secondary lung cancer absolute mortality risk at 30 years. The benefit of PBT was greatest among patients with concurrent axillary disease [3]. Though some may instead omit consolidation RT after a complete metabolic response for these larger targets including the mediastinum and axilla, randomized non-inferiority studies have demonstrated an inferior progression-free survival by 5–7% when RT is omitted among patients with early-stage disease [5,6].

Pencil beam scanning (PBS) PBT facilitates the treatment of larger volumes compared to passive scatter techniques. This is most evident in cranial spinal irradiation (CSI), in which gradient junctions enable the treatment of overlapping fields without the need for match lines [7,8]. Furthermore, the dose distribution is robust to potential variations in day-to-day patient setup along the region of overlap [8]. We hypothesized that PBS PBT may be an attractive modality for treating ETRT targets in HL patients. Using the Proton Collaborative Group (PCG) registry, we compare the dosimetric benefits of PBS PBT and modern photon techniques and describe the technical aspects of using PBS PBT for mediastinal and axillary targets.

## 2. Materials and Methods

### 2.1. Patient Cohort and Eligibility

We reviewed the records of all pediatric and adult HL patients in the PCG registry registered between 2016 and 2021. The PCG registry is a prospective, multi-institutional national registry of patients treated with PBT. Eligible patients included HL patients treated with PBT to both their mediastinum and at least one axillary region for whom the digital image communication in medicine of radiation-RT (DICOM-RT) was available by the time of data collection in November 2022. We extracted the size of the target volumes, table and gantry angles, and the number of isocenters. We also characterized the lowest extent of mediastinal disease using the left pulmonary artery (PA) and thoracic spine T8 as landmarks, as previously described [9]. Upper mediastinum was defined as disease above the level of the left pulmonary artery (PA); middle mediastinum disease was located below the left PA but at or above the level of the 8th thoracic spine vertebra (T8); and low mediastinum was disease below T8.

### 2.2. Toxicity Assessment and Follow-Up

Toxicity data were extracted from the PCG registry using the common terminology criteria for adverse events version 5 (CTCAE v5.0) [10]. We collected grade 3+ acute toxicities and all grades of radiation-associated pneumonitis. Patients were followed per the discretion of the treating radiation oncologists.

### 2.3. Comparative Volumetric Arc Therapy (VMAT) Plan

Dosimetric comparative photon plans were created and compared to the delivered PBT plans. Butterfly VMAT treatment planning was performed on the RaySearch RayStation treatment planning system V12A (RaySearch Laboratories, Stockholm, Sweden) and the treatments were planned with 2 partial arcs that spanned the anterior oblique (330°–30°) and posterior oblique angles (150°–210°), as previously described [11]. A non-co-planar (90° couch rotation) and an anterior partial arc (330°–30°) were also used in the optimization. All arcs were permitted to make double sweeps to improve target coverage. The photon plans were optimized so that the D (98%) of the planning target volume (PTV) in the VMAT plan was matched to the PTV D (98%) of the delivered proton plan. If the PTV D(98%) coverage in the photon plan could not match the PTV D (98%) of the proton plan, then a constraint of target coverage to the clinical target volume (CTV) or internal target volume (ITV) of D99 > 98% was used. If an ITV was present, the photon coverage was optimized to the ITV; however, if only a CTV was present (e.g., deep inspiratory breath hold (DIBH), no motion management), the CTV was used for target coverage. The VMAT organs at risk (OARs) prioritization was first the lungs, followed by the heart, and then the breast.

## 3. Results

### 3.1. Patient Demographics

Twelve patients were included in our study. Their median age was 26 years (range 17–54). Most (n = 9) were male, had stage 2 disease (n = 8), and were treated at initial diagnosis (n = 9). The remaining patients had either stage 3 (n = 1) or stage 4 (n = 3) disease. The most common histology was nodular sclerosing HL (n = 9). Seven (58%) received treatment to the unilateral axilla and five (42%) to both axillae. Eight (67%) patients also received radiation to the neck. All except one patient had either middle or low mediastinal involvement. Eight (66.7%) had lower, three (25%) had middle, and one (8.3%) had upper mediastinal disease (Table 1).

### 3.2. PBS Proton Therapy Characteristics

**Figure 1:**
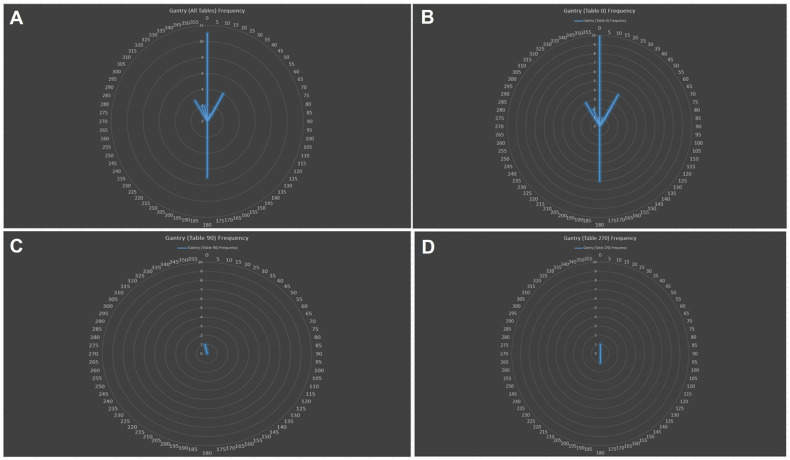
Frequency plot of gantry angles used for all delivered beams (**A**). Note that the AP beam is 0 and the PA beam is 180 degrees. Only 3 table positions were used, most frequently 0 (**B**) and less frequently 90 (**C**) and 270 degrees (**D**). Anterior oblique angles, when used, were typically only used for a table angle of 0.

**Figure 2:**
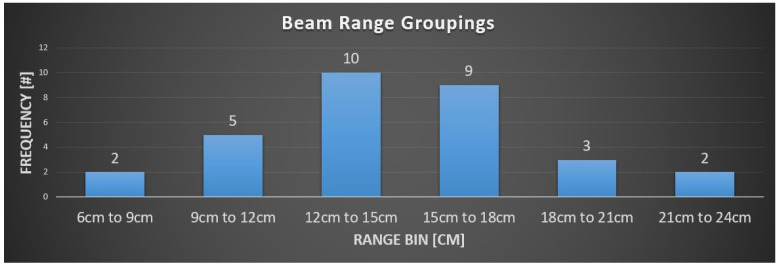
Histogram of beam ranges of beams delivered to targets.

The median dose was 30.6 Gy (RBE) (range 21–36). PBS was used in all patients. With regards to motion management, a free-breathing 4D CT scan was used in six (50%) patients and DIBH in one patient (8.3%). No motion management was used in two (16.7%) patients, and motion management status was unknown for the remaining three (25%) patients. Anterior (gantry 0) followed by posterior (gantry 180) beam angles were most frequently used, without a table kick (Figure 1). A minority of the beams used an anterior oblique angle, whereas no posterior oblique angles were used. Table kicks were infrequently used with anterior or posterior beams. A wide range of target depths were treated (Figure 2), likely reflecting the variable anatomic extent of the targets (e.g., neck/axilla vs. mediastinum). Most beams traveled between 12 and 18 cm to reach the treatment targets.

**Figure 3:**
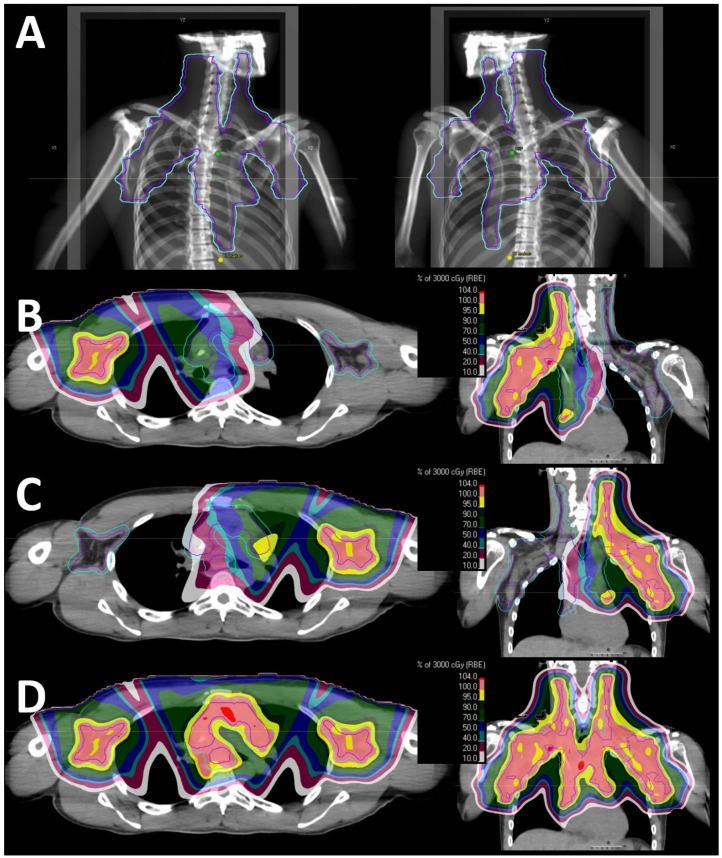
Proton beam therapy radiation plan for a patient treated with 2 isocenters. (**A**) Beam’s eye view (BEV) from anterior oblique angles, demonstrating that the left/right dimension of the clinical target volume is too large for a 30 × 40 cm snout, necessitating 2 isocenters. Beam-specific dose to the left isocenter (**B**), which includes the left axilla and partial mediastinum, and to the right isocenter (**C**), which includes the right axilla and partial mediastinum, and the resulting composite dose (**D**). Note that only one beam is used to treat each of the axillary targets, but two beams are used in the thorax, given the motion of the mediastinal target. There is an intentional overlap of the left and right isocenter fields in the mediastinum with a gradient junction. As such, no match line is needed. The following colored isodose lines represent the proportion (%) of the prescription dose (30 Gy (RBE)): red, 104%; coral, 100%; yellow, 95%; hunter green, 90%; forest green, 70%; navy, 50%; turquoise, 40%; burgundy, 20%; and white, 10%. The maximum hot spot was 105%.

All but two patients were planned with one isocenter. Both patients treated with >1 isocenter received radiation to the mediastinum, bilateral axillae, and bilateral neck; one also received radiation to the internal mammary lymph nodes. Though the numbers were small, patients treated with >1 isocenter had larger target volumes compared to patients treated with 1 isocenter: median PTV volume was 1455 vs. 1085 cc. Notably, the superior/inferior (SI) and left/right (LR) extent of the CTV/ITV was >30 cm in both patients treated with >1 isocenter; in contrast, patients treated with 1 isocenter had either SI or LR > 30 cm, but not both (Table 1). Figure 3 shows a representative treatment plan for a patient treated with two isocenters.

### 3.3. Dosimetry and Comparative Plans

As mentioned above, we attempted to match the photon PTV coverage to that achieved with PBT. However, in two patients, the PTV coverage was slightly compromised in the photon plans so that there would be adequate sparing of the heart and lungs: D(98%) was 95.9% (photons) versus 98.0% (PBT) for one patient and 76.4% versus 82.5% for the other patient. Despite similar or better PTV coverage, PBT was associated with lower median mean heart and lung doses (Table 2): mean heart, 9.03 Gy (RBE) [PBT] vs. 13.59 Gy [photons]; mean lung, 7.55 Gy (RBE) vs. 10.70 Gy. The median volumetric parameters of the lung were also lower with PBT compared to the photons: V5, 41.25% vs. 54.61%; V20, 15.11% vs. 24.81%. However, there was a similar dose to the breast across the V4 to V30 metrics, though the number of female patients was small (three patients). In addition, PBT was associated with better conformity and lower dose heterogeneity compared to butterfly VMAT.

### 3.4. Toxicity

Within a median follow-up of 24.6 months (range 6.7–60.1), no acute grade 3+ radiation-related toxicities were reported. Notably, no RT-associated pneumonitis was observed. One patient developed grade 1 pneumonitis 10 months after completing PBT and 4 months into ongoing immunotherapy. Given the timing after the initiation of immunotherapy and the imaging findings, this was attributed to immunotherapy. One patient developed a cough 2 weeks after starting RT. Given the timing, it was felt that this was unlikely to be RT-associated pneumonitis.

## 4. Discussion

Despite the irradiation of extensive targets spanning the mediastinum and axilla, PBS for ETRT was well tolerated, with no pneumonitis observed among these real-world cases. Indeed, our dosimetric comparison with advanced photon techniques demonstrated that PBS was associated with better target coverage, dose heterogeneity, and conformity, while also lowering the dose to the heart and lungs. Compared to other PBT techniques such as uniform scanning, PBS is more effective in sparing the proximal dose, which manifests as a lower dose to the lungs and skin. There is no control of the skin dose in uniform scanning or passive scattering, as the skin dose is purely a function of the field’s modulation and the skin depth itself within the field. In addition, the field matching demonstrated in Figure 3 could only be safely delivered using PBS delivery methods. We strove for fair dosimetric comparisons, specifically ensuring the same or better coverage of PBT compared to the VMAT plans before the dose to the OARs was pushed. Heart and lung sparing with PBS were especially noted across low (V5) to moderate (V20) doses, which have been associated with death (e.g., lung cancer mortality) among HL survivors [12]. The best available evidence suggests that the risk of coronary heart disease (CHD) is linearly related to the mean heart dose without a dose threshold [13]; that is, there is no dose threshold below which the risk of CHD is zero. Future studies are still needed to evaluate whether there is differential radiation sensitivity amongst the cardiac substructures.

Our findings validate those from Ntentas et al. [3], who observed a greater benefit of PBT over photon techniques among mediastinal lymphoma patients with axillary disease, given the greater reduction in mean lung dose and their resulting 30-year absolute mortality risk of lung cancer. Among the three female patients in our cohort, PBS did not appear to have a dosimetric advantage over VMAT in breast sparing. The treatment of the axilla entails the coverage of more breast tissue given the co-localization of the targets. While an inclined breast board may pull the breast tissue inferiorly, it should be noted that nearly all the patients had middle or lower mediastinal involvement, which would also bring the co-planar dose lower when these intra-thoracic targets are irradiated.

We observed that, for some cases, VMAT was unable to yield a plan with a “safe” dose to the lung, reflecting the dosimetric superiority of PBS. The risk of radiation pneumonitis (of any grade) is about 10% in consolidation and 25% in relapsed/refractory settings. Dosimetric predictors of pneumonitis have been identified amongst lymphoma patients treated with 3D, conformal [14], intensity-modulated radiation therapy (IMRT) [15] and proton therapy [16], with similar findings. Notably, mean lung doses of >13.5 Gy, V20 of >33.5%, and V5 of >55% were associated with an increased risk of radiation pneumonitis [14,15,16]. While nearly all the PBT patients’ plans met these lung dose constraints, the median lung V5 from the comparative VMAT plans was 55%; that is, half of the patients had doses greater than the lung constraint that has been the strongest predictor of pneumonitis among patients treated with IMRT [15]. The ability of PBS plans to meet the lung constraints is validated clinically by a very favorable subacute toxicity profile, in which no RT-associated pneumonitis was noted within this prospective registry. All together, these findings suggest that lymphoma patients who require both mediastinal and axillary treatment are another subgroup of lymphoma patients who may benefit from PBS PBT. As our cohort did not include patients treated with uniform or passive scattering, it is unclear to what extent dosimetric sparing may be achieved with aperture- and compensator-based PBT techniques compared to modern photon techniques. A recent report from the lung PCG registry showed a lower probability of pulmonary toxicity with PBS compared to older techniques (e.g., passive scattering and uniform scanning) (0.08 vs. 0.34, respectively) among locally advanced lung cancer patients, suggesting that there may be differential lung sparing across PBT techniques [17].

Arguably, the best lung sparing with photons may be achieved with a 3D conformal technique (e.g., AP:PA), but at the cost of less heart sparing. Indeed, the most common proton beam angles used within this cohort are anterior beams, with or without a posterior beam. Given the rapid dose drop-off along the beam path, PBT, and in particular PBS, has the unique ability to also carve the dose around the heart (i.e., to achieve both heart and lung sparing).

Similar patterns appear to emerge from this study of “real-world” delivered PBS ETRT plans. Anterior beams, with or without a posterior beam, may serve as reasonable starting beam arrangements. Anterior oblique angles can be considered. Assuming a 30 × 40 cm snout, most CTV or ITV targets with an SI and/or LR < 30 cm can typically be accommodated for with a single isocenter. Targets with an SI and LR > 30 cm may require >1 isocenter if the target volume length is larger than the snout. PBS plans requiring >1 isocenter are relatively easy to deliver given the gradient junction between the two fields, which does not require a match line (Figure 3).

Several limitations should be noted. Despite modest patient numbers, our report on 12 patients with extensive clinical and dosimetric information is a valuable addition to the literature. The small numbers here, despite being drawn from a multi-institutional registry, may reflect the rarity of mediastinal and axillary targets (less likely), or patients having RT deferred given toxicity concerns. As less breast sparing can be achieved with ETRT compared to a mediastinal-only target, additional consideration should be made with respect to the patient’s age, sex, and disease status (e.g., a complete metabolic response versus partial metabolic response). Indeed, this may in part explain the predominance of males within our cohort. Additional limitations of this study include a lack of information on the treatment planning algorithms that were used for PBT planning. Therefore, the coverage of the target volume may be smaller and the heterogeneity of the delivered PBT plans may be greater than calculated if a pencil beam algorithm were used instead of a Monte Carlo algorithm [18]. The patient data acquired for this study were obtained from a registry submission. Dose coverage constraints varied across the patient cohort and were institutionally dependent. In photon-based planning, PTV coverage is universally accepted as the appropriate index of target coverage plan quality. In proton therapy, plan quality, with regards to target coverage, is typically evaluated using a robust evaluation of the CTV/ITV coverage in the presence of set-up errors and proton range uncertainty, and PTV coverage is considered irrelevant. For these reasons, it is often challenging to make direct target coverage equivalences between proton and photon plan quality. In this study, photon plans were required to meet the PTV coverage of the clinically treated proton plans and may not have been considered clinically acceptable within a photon environment. Lastly, though all patients were followed for toxicity, follow-up was not standardized. Specifically, imaging may not have been routinely performed after PBT. As such, grade 1 pneumonitis (e.g., radiographic evidence) in clinically asymptomatic patients may have been missed.

## 5. Conclusions

Proton ETRT can be safely delivered with PBS and is associated with an improved sparing of the heart and lungs compared to modern photon techniques. Patients requiring both mediastinal and axillary treatment may be another subgroup of lymphoma patients who may benefit from PBT.

## Figures and Tables

**Table 1:** Characteristics of patient plans with one and >one isocenter.

Characteristics	All Patients (n = 12)	One Isocenter (n = 10)	>One Isocenter (n = 2)
Neck Involvement	8 (66.7%)	6 (60%)	2 (100%)
Unilateral	2 (16.7%)	2 (20%)	0
Bilateral	6 (50%)	4 (40%)	2 (100%)
Mediastinum Involvement	12 (100%)	10 (100%)	2 (100%)
Upper	1 (8.3%)	1 (10%)	0
Middle	3 (25%)	3 (30%)	0
Lower	8 (66.7%)	6 (60%)	2 (100%)
Axilla	12 (100%)	10 (100%)	2 (100%)
Unilateral	7 (58%)	7 (70%)	0
Bilateral	5 (42%)	3 (30%)	2 (100%)
Internal Mammary Lymph Node Involvement	2 (16.7%)	1 (10%)	1 (50%)
Hila/Hilum Involvement	1 (8.3%)	1 (10%)	0
Largest CTV/ITV Dimension (Median, Range)			
Left/right	22.75 (15.9–38.7)	21.6 (15.9–38.7)	32.2 (31.4–33)
Superior/inferior	22.2 (13.6–36.3)	19.35 (13.6–29)	34.05 (31.8–36.3)
PTV (Median, Range)			
Volume (cc)	1197.84 (257.44–1834.54)	1162.12 (257.44–1834.54)	1454.61 (1117.92–1791.3)

**Table 2:** Dosimetry comparison between VMAT photon and PBS proton plans.

DVH Parameter (Median, Range)	Photon	Proton
Heart		
Mean (Gy (RBE))	13.59 (2.77–24.88)	9.03 (2.36–12.85)
V5	58.68% (9.62–94.53)	43% (9.48–65.06)
V10	48.67% (6.07–85.02)	35.19% (7.18–54.97)
V15	43.51% (4.31–75.38)	29.50% (5.63–46.42)
V20	34.76% (2.94–66.46)	23.33% (4.57–35.55)
V30	7.04% (0–46)	6.06% (0–21.17)
Lung		
Mean (Gy (RBE))	10.7 (6.56–18.57)	7.55 (4.07–13.63)
V5	54.61% (35.96–91.95)	41.25% (26.37–56.63)
V10	39.58% (25.33–64.2)	30.42% (15.95–44.4)
V15	33.49% (18.45–54.43)	21.48% (8.64–37.81)
V20	24.81% (4.00–47.47)	15.11% (3.55–32.31)
V30	4.51% (0–28.07)	2.43% (0–20.31)
Thyroid		
Mean (Gy (RBE))	28.54 (1.59–32.21)	26.42 (17–31.92)
V5	100% (0–100)	99.10% (0–100)
V10	100% (0–100)	94.24% (0–100)
V15	98.36% (0–100)	90.39% (0–100)
V20	99.41% (0–123.03)	83.4% (0–100)
V25	88.09% (0–100)	71.35% (0–100)
V30	50.34% (0–98.48)	41.43% (0–99.85)
Breast *		
Mean (Gy (RBE))	3.47(1.49–9.68)	3.15 (1.13–6.86)
V4	20.94% (10.67–47.12)	23.41% (10.56–33.39)
V5	18.27% (9.38–45.15)	21.97% (9.72–31.96)
V10	11.11% (4.3–36.89)	16.04% (3.15–26.66)
V15	7.4% (1.81–27.85)	5.11% (0.66–22.79)
V20	5.26% (0.02–22.4)	2.88% (0.01–19)
V25	3.29% (0–16.81)	1.4% (0–12.69)
V30	0.41% (0–7.31)	0.43% (0–2.44)
ITV/CTV		
Volume (cc)	634.68 (131.7–1074.23)	634.68 (131.7–1074.23)
Mean (Gy (RBE))	31.65 (21.74–46.52)	31.26 (21.38–45.71)
Mean (as % of prescription)	100% (100–103.5)	102% (101.3–106.5)
D(98%) (as % of prescription)	96.6% (94.4–100)	100% (98.4–100.7)
D(99%) (as % of prescription)	95.9% (93.3–99.7)	99.2% (97–100.5)
D(99.9%) (as % of prescription)	93.9% (87–98.8%)	97.7% (89.5–100)
PTV		
Volume (cc)	1197.84 (257.44–2834.54)	1197.84 (257.44–2834.54)
Mean (Gy (RBE))	31.31 (21.37–46.01)	31.07 (21.14–45.54)
Mean (as % of prescription)	99% (98.3–101.3)	101% (100–104)
D(98%) (as % of prescription)	91.1% (76.4–97.7)	93.45% (82.5–98.9)
D(99%) (as % of prescription)	88.3% (65.6–96.5)	91.3% (76.5–97.8)
D(99.9%) (as % of prescription)	75.77% (34.7–89.6)	80.6% (56.8–95)
Heterogeneity Index		
(D (2%) − D (98%))/D (50%)	0.14 (0.07–0.32)	0.11 (0.05–0.25)
Conformity Index **	1.44 (1.05–1.75)	1.09 (1.01–1.43)

All values are medians (range); * breast dose was calculated in the 3 female patients; ** treated volume enclosed by 100% dose/Vol in PTV receiving 100% dose.

## Data Availability

The data presented in this study are available on request, if reasonable, from the corresponding author.

## Abbreviations

BVMAT: butterfly volumetric arc therapy; CSI: cranial spinal irradiation; CTCAE v5.0: common terminology criteria for adverse events version 5; CTV: clinical target volume; DIBH: deep inspiratory breath hold; DICOM-RT: digital image communication in medicine of radiation-RT; ETRT: extended target radiation therapy; HL: Hodgkin lymphoma; IMRT: intensity-modulated radiation therapy; ITV: internal target volume; ILROG: International Lymphoma Radiation Oncology Group; LR: left/right; OARs: organs at risk; PA: pulmonary artery; PBS: pencil beam scanning; PBT: proton beam therapy; PCG: Proton Collaborative Group; PTV: planning target volume; SI: superior/inferior; VMAT: volumetric arc therapy.
